# Technofunctional Properties and Proteomic Profiling of Sesame Cake Protein Isolates Obtained by Green Extraction Methods and Treated with Cold Plasma

**DOI:** 10.3390/molecules31152638

**Published:** 2026-07-29

**Authors:** Emine Gizem Acar, Ozge Nur Ozturk, Celale Kırkın, Ekin Sonmez, Huseyin Cimen, Derya Kahveci

**Affiliations:** 1 Department of Food Engineering, Faculty of Chemical and Metallurgical Engineering, Istanbul Technical University, 34469 Istanbul, Türkiye; acaremi17@itu.edu.tr (E.G.A.); kirkin@itu.edu.tr (C.K.); 2 The Institute of Biotechnology, Gebze Technical University, 41400 Kocaeli, Türkiye; o.ozturk2025@gtu.edu.tr (O.N.O.); ekinsonmez@gtu.edu.tr (E.S.); 3 Central Research Laboratory Application and Research Center (GTUMAR), Gebze Technical University, 41400 Kocaeli, Türkiye; 4 Zero Waste Institute, Istanbul Technical University, 34469 Istanbul, Türkiye

**Keywords:** sesame cake protein, cold plasma, technofunctional properties, proteomics, allergenicity

## Abstract

The demand for plant-based proteins has been steadily increasing; however, their functionality when applied in food product formulations requires comprehensive evaluation and, when necessary, deliberate modification to ensure that it mimics that of animal-derived proteins. In the present study, protein was extracted from sesame cake, a by-product of sesame oil production, through conventional alkaline extraction, with or without ultrasound or enzymatic pretreatments. Cold plasma treatment was subsequently applied to sesame cake proteins (SCPs) to induce structural modifications. Both the extraction method and cold plasma treatment markedly influenced the compositional and technofunctional properties of the SCPs, including the protein purity, amino acid profile, color, structural integrity, thermal stability, solubility, emulsifying capacity, foaming behavior, and proteomic profile. Importantly, combined ultrasound and cold plasma treatments altered the proteomic landscape, leading to the downregulation of proteins associated with allergenicity and thereby mitigating the adverse effects of SCPs. Notably, this study provides the first demonstration of the interplay between the extraction method and protein responsiveness to cold plasma treatment.

## 1. Introduction

Food security remains a major challenge to be solved by the global community; 512 million people are expected to suffer from chronic undernourishment by 2030, which presents a significant obstacle to the achievement of Sustainable Development Goal 2, Zero Hunger [1]. Protein intake, either animal- or plant-based, is an important component of tackling undernourishment; plant-based proteins are on the rise, since animal-based options have been shown to have a negative impact on the environment and are less attractive to consumers, especially those who are sensitive to the sustainability aspects of their production, as well as animal welfare [2,3]. Oilseed press cake, which is what remains after oil extraction from seeds using an expeller, is a major by-product of the edible oil industry, together with the oilseed meal obtained after the additional solvent processing of the cake. Due to their enriched protein concentrations, these by-products have long been of interest as a raw material for plant-based protein production [4].

Sesame (*Sesamum indicum* L.) is an ancient oilseed crop with a 45–50% lipid content and a 20–25% protein content. The cold-press extraction of oil from sesame seed is the most widely used process, and the by-product of sesame oil production, sesame cake, is either discarded or used as animal feed [5]. However, since the remaining sesame cake is enriched in protein (up to 50%), current attempts focus on the valorization of this by-product for protein extraction [6]. The amino acid profile of sesame cake protein (SCP) provides the FAO/WHO/UNU essential amino acid nutritional requirements for adults, such that its dietary use is promising, especially given its content of methionine and cysteine, which are often lacking in plant-based sources, while lysine is the first limiting amino acid [7]. SCP has been shown to be widely applicable in food products, with an emulsifying activity and stability comparable to, if not exceeding, those of soy and pea proteins [6], leading to its successful application in meatball analogs [8]. The emulsification potential of SCP was further improved by physical treatments, such as ultrasound [9] and high-pressure homogenization [10]. The foaming properties of SCP were shown to be impressive as well [11], with improvements demonstrated that were similar to those achieved using the abovementioned treatments [9,10]. These properties of SCP enabled its use in foam-type products, such as gluten-free rice cakes, which showed good nutritional as well as sensory properties [12].

Several approaches are available for the extraction of protein, where alkaline extraction followed by isoelectric precipitation (called conventional extraction from this point forward) is the most widely used one when plant-based sources are used as the raw material. However, this method attracts some criticism due to the high volumes of alkaline and acidic solutions used, as well as the poor functionality of the protein concentrate obtained [13]. Therefore, novel methods such as enzyme and ultrasound pretreatments have been investigated as alternatives, either alone or in combination. Enzyme pretreatment facilitates the loosening of rigid fibrous structures and the breakdown of protein–carbohydrate interactions, thereby improving the release of proteins in the following aqueous extraction step [14]. Applications of protease- and/or carbohydrase-based enzyme preparations have been shown to enhance the yield of protein extraction through the conventional extraction from oilseed cakes [14,15,16], in addition to other benefits; for example, the authors of [17] reported that the pectinase treatment of rapeseed cake enabled the water extraction of proteins without further pH adjustment. Improved functional properties due to the enzyme-aided modification of the protein structure, such as solubility [15,16,18], gelation [14], antioxidant capacity [19,20], emulsifying activity [19], and foaming stability [21], have been reported as well. Ultrasound pretreatment, where acoustic cavitation leads to the disruption of networks of hydrogen and disulfide bonds and thereby alters the secondary and tertiary structures of proteins [22], has also been applied to assist conventional extraction from plant-based sources with improved protein recovery [23], digestibility [24], foaming capacity, and stability [23]. Ultrasound has the important advantage of shortening the extraction time, where the extent of the structure and functionality modification has been shown to be dependent on treatment conditions, especially the energy density (the amount of ultrasound energy applied per unit volume of sample slurry) [25]. Sonication applied to sesame meal protein post-extraction was also sufficient to modify its technofunctional properties [26].

Given the well-established relationship between the protein structure and technofunctional and nutritional properties, the pursuit of effective strategies for protein modification has long been a central focus of research. Heat and/or chemical treatment-induced modifications to protein have been replaced by non-thermal options in an attempt to provide energy-efficient and chemical-free routes to develop proteins with fine-tuned properties, where cold plasma application stands out for being an environmentally friendly, uniform treatment that does not require solvents and has a short processing time and low installation and implementation costs [27]. Reactive species generated by the ionization of gas (mostly atmospheric air in the current literature) due to electrical energy applied, leading to the generation of plasma as the fourth stage of matter, are responsible for protein modification via bond cleavage and oxidative reactions [28]. Several oilseed proteins, such as soybean [29,30], sesame [31,32], sunflower [31], and hazelnut [33] proteins, have been treated using cold plasma to successfully modify their structure and thereby their technofunctional properties.

The selected route for protein extraction has been shown to influence several structural and physicochemical properties of the end product, including the amino acid and phenolic concentrations, solubility, digestibility, emulsifying and foaming properties, and water and oil holding capacity [13,34,35]. Cold plasma processing parameters, on the other hand, such as treatment time and voltage, also affect the extent of structural modification [27]. However, how the extraction method used for a given plant protein influences its subsequent response to cold plasma treatment remains largely unexplored. Therefore, the aim of this work was to evaluate three protein extraction approaches, namely, alkaline extraction followed by isoelectric precipitation (referred to as conventional extraction), either alone or combined with enzymatic or ultrasound pretreatments, to produce sesame cake protein isolates. These isolates were subsequently compared regarding their technofunctional properties before and after cold plasma treatment, aiming to elucidate the relationship between the extraction approach and the efficacy of cold plasma in driving structural and proteomic alterations.

## 2. Results

### 2.1. Extraction of Sesame Cake Protein (SCP)

**Figure 1:**
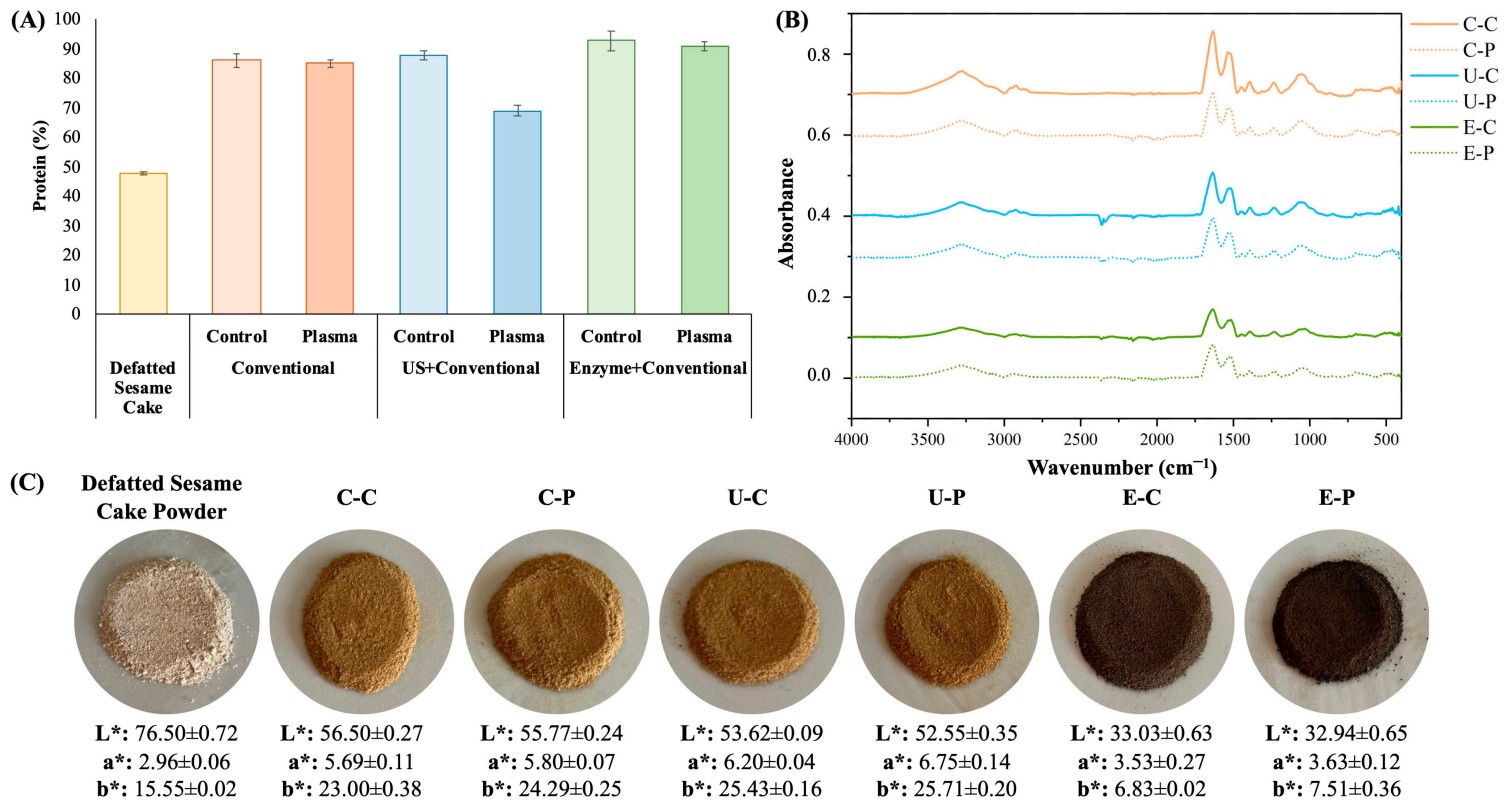
The influence of extraction and cold plasma treatment on SCPs: (**A**) protein contents of defatted sesame cake and SCPs obtained by conventional extraction, with or without ultrasound and enzymatic pretreatments; (**B**) FTIR spectra of SCPs between 4000–400 cm^−1^; and (**C**) color parameters of defatted sesame cake powder and SCPs. Sample codes: C, U, and E stand for conventional, ultrasound-pretreated, and enzyme-pretreated, respectively. C and P stand for control (not treated) and cold plasma treated, respectively.

SCPs were obtained via conventional extraction with or without ultrasound and enzymatic pretreatments, and their protein purities were given in Figure 1A. All extraction methods yielded a protein purity exceeding 85% (Figure 1A). Cold plasma treatment significantly reduced the protein content of SCPs obtained with ultrasound pretreatment, whereas this effect was not observed for the other extraction methods. The extraction yield (ranging from 11.75 ± 0.35 to 12.64 ± 0.74) was not affected by the extraction method (*p* > 0.05); however, pretreatments, especially ultrasound, significantly increased protein recovery from 21.20 ± 0.56% up to 23.28 ± 0.43% (*p* < 0.05).

### 2.2. Amino Acid Profile of SCPs

All 16 amino acids present in the standard mixture and detectable by OPA derivatization were identified across all SCP samples, with no lacking amino acids (Table 1). The extraction method had no significant effect on the amino acid profile (*p* > 0.05). Although individual amino acid levels were not significantly altered, cold plasma treatment had a significant negative impact on the essential amino acid concentration (*p* < 0.05).

### 2.3. Physicochemical Properties of SCPs

#### 2.3.1. Structural Characterization

The FTIR spectra of SCPs were recorded in the range of 4000–400 cm^−1^ (Figure 1B). The prominent peaks were all the same in each SCP, which were around 3200, 2900, 1700–1500, and 1200–1000 cm^−1^. Cold plasma treatment altered the structural peaks of proteins, generally resulting in a decrease in the peak intensity.

#### 2.3.2. Thermal Properties

The thermal degradation characteristics of SCPs were evaluated using differential scanning calorimetry (DSC), and the properties of denaturation peaks were summarized in Table 2. The lowest onset and maximum temperatures were observed in SCPs obtained via conventional extraction without cold plasma treatment (C-C). In contrast, applying cold plasma to these proteins (C-P), as well as the incorporating enzymatic or ultrasound pretreatments during extraction (E-C and U-C), significantly increased both temperatures, indicating an enhanced thermal stability (*p* < 0.05).

#### 2.3.3. Color

The color of the enzymatically pretreated SCPs, E-C and E-P, were significantly different from the rest (Figure 1C). Cold plasma treatment was found to have a significant effect on all L*, a*, and b* values (*p* < 0.05). The ΔE values calculated with respect to before and after cold plasma treatment were found to be 1.64 ± 0.16, 0.92 ± 0.16, and 1.04 ± 0.14 for SCPs coded C, U, and E, respectively.

### 2.4. Technofunctional Properties of SCPs

#### 2.4.1. Protein Solubility

**Figure 2:**
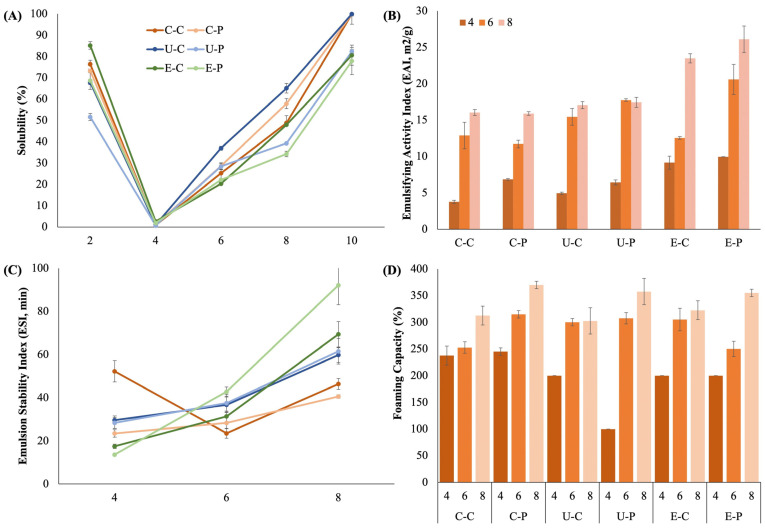
Technofunctional properties of sesame cake proteins across pH ranges: (**A**) protein solubility of SCPs in a pH range of 2–10; (**B**) emulsifying activity index (EAI, m^2^/g) and (**C**) emulsion stability index (ESI, min) of SCPs in a pH range of 4–8; and (**D**) water and oil holding capacity (WHC and OHC) of SCPs in a pH range of 4–8.

The protein solubility (%) of SCPs was evaluated across a pH range of 2 to 10 (Figure 2A). All protein samples exhibited minimum solubility, approaching zero, at pH 4, which represents the isoelectric point. All factors (extraction method, cold plasma treatment, and pH) had a significant effect on protein solubility (*p* < 0.05). Compared to the conventional method, applying enzymatic and ultrasound pretreatments significantly decreased protein solubility (*p* < 0.05). Although the solubility trends across the pH range were similar across different extraction methods, cold plasma treatment increased the solubility of SCPs produced by conventionally and ultrasound-pretreated extraction, whereas it significantly reduced the solubility of enzyme-pretreated SCPs (*p* < 0.05).

#### 2.4.2. Emulsifying Properties

The emulsifying properties of SCPs were first determined spectrophotometrically in terms of the emulsifying activity index (EAI) and emulsion stability index (ESI) (Figure 2B,C). All factors significantly affected the emulsifying activity (*p* < 0.05). While cold plasma treatment had a significant effect on the EAI (*p* < 0.05), its effect on stability was not statistically significant (*p* > 0.05). Both the EAI and ESI values increased as the pH rose from 4 to 8. SCPs obtained via conventional extraction exhibited the lowest overall emulsifying properties, whereas enzymatic pretreatment proved more effective than ultrasonic pretreatment in enhancing the EAI (*p* < 0.05).

Furthermore, the droplet size and zeta potential of oil-in-water emulsions stabilized with SCPs were evaluated and summarized in Table 3. Droplet sizes (nm), reported as the z-average and number mean, were both significantly influenced by the extraction method, cold plasma treatment, and pH (*p* < 0.05). The largest droplet size, which indicates lower emulsifying activity, was observed when SCPs obtained via ultrasonic pretreatment were used, followed by conventional extraction. Transitioning the pH from acidic to alkaline conditions, and, more notably, applying cold plasma treatment, significantly reduced the emulsion droplet size (*p* < 0.05). Although the zeta potential was not significantly affected by cold plasma treatment (*p* > 0.05), it varied considerably as the pH increased (*p* < 0.05). Consistent with their smaller droplet sizes, SCPs extracted with enzymatic pretreatment exhibited the highest zeta potential (*p* < 0.05).

#### 2.4.3. Water and Oil Holding Capacities

The extraction method significantly affected both the water holding capacity (WHC) and oil holding capacity (OHC) of SCPs, whereas cold plasma treatment had a significant effect only on the OHC (*p* < 0.05). No consistent trend was observed for SCPs obtained via enzymatic or ultrasound pretreatments; however, cold plasma treatment significantly increased the OHC and decreased the WHC in conventionally extracted SCPs (Table 4).

#### 2.4.4. Foaming Capacity and Stability

The foaming capacity (FC) of SCPs depended on both the extraction method and pH (Figure 2D). The FC decreased under acidic conditions, but increased under increasingly alkaline environments (*p* < 0.05). While cold plasma treatment had no significant effect on pretreated SCPs, it markedly enhanced the FC of conventionally extracted SCPs, achieving increases of up to 25%. Over a 60-min monitoring period, cold plasma treatment significantly improved the foam stability (FS) across all time intervals (Table 5). Among the extraction methods, ultrasound pretreatment yielded the highest FS, whereas conventional extraction at pH 6 produced the lowest.

### 2.5. Molecular Weight Distribution and Proteome Analysis

**Figure 3:**
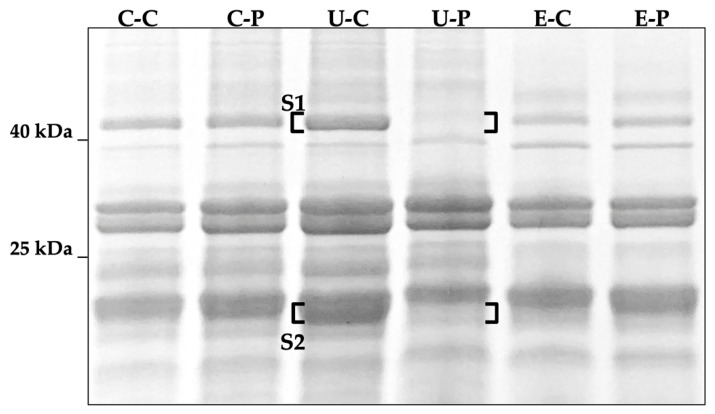
SDS-PAGE analysis of SCPs. Molecular weight markers indicate 40 kDa (S1) and 25 kDa (S2), while brackets highlight bands selected for in-gel trypsin digestion and subsequent LC-MS/MS analysis.

An SDS-PAGE analysis of SCPs obtained via conventional, ultrasound-pretreated, and enzyme-pretreated extraction revealed distinct proteomic profiles of the different methods and treatments (Figure 3). Dominant bands were observed in the ~40 kDa and ~20 kDa regions in all lanes. Specifically, two bands from the ultrasound-pretreated samples exhibited distinct differences when compared to the conventional control (U-C vs. C-C) as well as following cold plasma treatment (U-P vs. C-P). Therefore, these bands were selected for in-gel tryptic digestion, followed by Liquid Chromatography-Tandem Mass Spectrometry (LC-MS/MS) analyses. Band S1 corresponds to the excised ~40 kDa region, while band S2 corresponds to the excised ~20 kDa region.

Protein identifications were performed against the *Sesamum indicum* reference within the UniProt Knowledge Database at a false discovery rate (FDR) threshold of 5%, requiring at least one unique peptide per protein identification. The primary identified proteins, 11S globulin seed storage protein 2, Peroxiredoxin-1, and 60S ribosomal protein L12-3, are summarized in Table 6 along with their UniProt accession numbers, theoretical molecular weights, peptide counts, and representative peptide sequences.

## 3. Discussion

### 3.1. Extraction of Sesame Cake Protein (SCP)

The SCPs exhibited a high protein purity across all three extraction methods, consistent with previous findings by Nouska et al. [35] and Raei et al. [36]. The protein content of SCPs even exceeded that reported for sesame cake protein extracted using cold plasma [37]. Incorporating pretreatments further enhanced the protein content from 85.92% to 87.65% with ultrasound and 92.57% with enzymatic pretreatment. Similarly, these pretreatments improved both the protein recovery from the sesame cake and overall extraction yield. This observation was expected, since carbohydrases facilitate protein extraction by hydrolyzing plant cell wall polysaccharides, whereas ultrasound promotes cell disruption and mass transfer, accelerating protein release and improving extraction efficiency [38].

Although cold plasma treatment was reported not to alter the protein content of wheatgrass flour [39], our results demonstrated a decrease in the protein purity of SCPs (Figure 1A). This reduction may stem from the reactive oxygen and nitrogen species generated during plasma exposure, which alter protein side chains and disrupt intermolecular interactions, thereby driving molecular rearrangement [40]. While protein digestibility was not evaluated in this study, the observed decrease in measured protein purity does not necessarily imply a reduced nutritional value; in fact, protein bioaccessibility may have improved. Cold plasma can fragment or degrade protease inhibitors by modifying their amino acid side chains, potentially enhancing the overall plant protein digestibility [28]. Achieving such improvements requires the precise optimization of cold plasma processing conditions. Moderate protein oxidation can increase proteolytic sensitivity by exposing previously hidden peptide sites, while excessive oxidation impairs enzymatic digestibility by inducing protein aggregation and covalent cross-linking [41].

### 3.2. Amino Acid Profile of SCPs

Regardless of the extraction method or cold plasma treatment, glutamic acid and aspartic acid were the most abundant amino acids in all SCPs, followed by leucine and arginine. The predominance of acidic amino acids aligns with previous studies reporting typical amino acid profiles for sesame proteins [6,36,42]. This high proportion of glutamic and aspartic acids is advantageous for bioactivity, as their side chains containing carboxyl and amine groups facilitate metal ion chelation and confer strong antioxidant properties via electron donation and free radical scavenging [43]. Furthermore, all SCPs met the FAO/WHO-recommended baseline of at least 40% essential amino acids relative to the total amino acid content, displaying values between 44 and 48%. These results suggest that SCPs serve as a high-quality protein source.

Regarding the impact of the extraction method on the amino acid profile, the highest total amino acid content was recorded in SCPs obtained via enzymatic pretreatment. This outcome can be attributed to the catalytic action of the enzyme used (Viscozyme), a mixture consisting of a wide variety of carbohydrases, which hydrolyzes the polysaccharide matrix of the cell wall and releases intracellular protein fractions [44].

The degradation of proteins or the loss of specific amino acid residues alongside the relative stability of lysine during cold plasma treatment is consistent with findings reported for various animal and plant proteins [41]. Cold plasma treatment induced a significant reduction in both essential and total amino acid contents. This can be explained by the reactive oxygen or nitrogen species generated during cold plasma treatment, which induce partial denaturation, particularly targeting sulfur-containing structures [45]. Hydroxyl radicals generated during treatment can oxidatively cleave disulfide bonds, destabilizing the protein structure and exposing free sulfhydryl groups [30]. Furthermore, reactive oxygen and nitrogen species drive protein carbonylation through the irreversible oxidation of amino acid side chains [46]. Interestingly, no reduction in total amino acid content was observed in enzyme-pretreated SCPs, suggesting an increased resistance to reactive species. This resistance may be attributed to partially oxidized polyphenols generated during enzymatic pretreatment, which can interact with proteins through covalent bonding and form protein–polyphenol complexes. Such complexes enhance antioxidant activity [47], thereby safeguarding SCPs against further oxidative degradation (Figure 1A).

### 3.3. Physicochemical Properties of SCPs

#### 3.3.1. Structural Characterization

FTIR spectroscopy is widely used to identify protein functional groups, evaluate secondary structure and side-chain characteristics, and detect conformational changes and protein purity. Due to the high protein purity of SCPs, peaks corresponding to protein backbone structures dominated the FTIR spectra (Figure 1B). All SCPs displayed major absorbance bands near 3280 cm^−1^ for Amide A (OH and amine stretching vibrations) and 2920 cm^−1^ for Amide B (CH_2_ asymmetric stretching) [8,48]. The peak at 1635 cm^−1^ observed in the Amide I region (1700–1600 cm^−1^), present across all samples, is assigned to C=O stretching vibrations of the peptide bond [24]. The Amide I band reveals the secondary structure of proteins, with the 1635 cm^−1^ peak indicating the presence of β-sheets [9,49]. The peak at 1520 cm^−1^ corresponds to the intermolecular/intramolecular hydrogen bonds within the Amide II region (1480–1575 cm^−1^) [24]. The peak at 1230 cm^−1^ in the Amide III region (1200–1400 cm^−1^), another characteristic protein transmission band, indicates the C–N stretching and N–H bending vibrations [24]. Finally, the peak observed at 1055 cm^−1^ indicates C–O stretching vibrations (Figure 1B) [15].

While the highest peak intensities across these wavelengths was observed in conventionally extracted SCP, the band intensities decreased in samples subjected to ultrasonic and enzymatic pretreatments. This reduction can be explained by the physical interactions during the pretreatments that alter the secondary structure of the protein by disrupting molecular interactions, driving molecular rearrangement, and promoting the formation of new bonds [48,50]. Although the number and position (wavenumber) of the primary protein peaks remained unchanged, a decrease in peak intensity was observed after cold plasma treatment, particularly in conventionally extracted SCP. Reactive oxygen and nitrogen species induce oxidative modifications in the primary structure of proteins, triggering structural rearrangements within the secondary and tertiary networks and the development of more randomized conformations [41]. The smaller peaks obtained compared to the control likely reflect the folding differences in the secondary structures caused by these plasma-generated reactive species (Figure 1B).

#### 3.3.2. Thermal Properties

Since thermal processing is widely used in food manufacturing, the thermal properties of the ingredients used are of critical importance. The denaturation temperatures of SCPs ranged from 123.47 ± 6.04 °C to 170.01 ± 0.42 °C, with conventionally extracted SCP displaying the lowest value. In comparison, the reported denaturation temperatures for sesame protein isolates in the literature are notably lower: 116.48 °C by Farhan et al. [8], 113 °C by Yang et al. [51], and 102 °C by Nouska et al. [35]. The higher denaturation temperatures observed in this study indicate superior thermal stability. Indeed, the maximum temperatures increased to 147.50 ± 2.18 °C for ultrasound-pretreated SCP (U-C), and to 160.59 ± 6.12 °C in enzyme-pretreated SCP (E-C), demonstrating that the applied pretreatments before extraction significantly enhanced the thermal stability of the SCPs.

Cold plasma treatment exerted contrasting effects on thermal stability depending on the extraction route: it significantly increased the denaturation temperature of conventionally extracted SCP, whereas it reduced the thermal stability of both U-C and E-C samples. The effect of cold plasma on protein thermal stability varies depending on factors such as modifications to the hydrophobicity, secondary structure configuration (α-helix and β-sheet), hydrogen bonding strength, and overall molecular conformation [52]. The enhanced thermal stability observed in plasma-treated conventionally extracted SCP can be attributed to plasma-induced protein aggregation and cross-linking [53]. However, the decrease in bond absorption density observed in the protein’s structure due to enzyme and ultrasound pretreatment may have rendered the proteins more susceptible to further structural disruption by cold plasma treatment, thereby lowering their thermal stability (Figure 1B). Cold plasma often impairs the thermal stability of proteins by disrupting intramolecular interactions through reactive-species-induced oxidation and structural modification, which leads to a lower denaturation enthalpy and reduced resistance to thermal unfolding [28,54]. Nevertheless, even following plasma-induced decreases, the denaturation temperatures of the control (non-treated) SCPs remained high. Because many food processes operate below these thresholds, these SCPs remain highly suitable for integration into food formulations.

#### 3.3.3. Color

The color of SCP reported by Nouska et al. [35] was lighter than all SCPs produced in the present study, but similar to the initial raw material, defatted sesame cake powder. The color parameters of the conventionally extracted SCPs closely matched those reported in a similar study by Mathews et al. [24]. The darker color with nearly identical L*, a*, and b* values of enzyme-pretreated SCPs aligned with the SCP obtained via microwave-assisted extraction in the same study. The authors explained the darker color by the electrochemical damage of microwaves. Nevertheless, the reduced lightness, green, and yellow values after enzymatic pretreatment can be attributed to the degradation of natural pigments and the concomitant release of other compounds, such as polysaccharides, into the extraction medium [55]. Furthermore, the enzymatic breakdown of the cell wall facilitates the release of bound phenolics, which are susceptible to partial oxidation by enzymes or oxygen in the environment. The oxidation of phenolic compounds generates quinones, which condense into brown pigments [56].

The ΔE value represents the color difference between samples (Figure 1C). In this study, it was evaluated to determine whether cold plasma treatment significantly affected the color of the protein isolates. The minimum color change was observed in ultrasound-pretreated SCP, followed by enzyme-pretreated and conventionally extracted SCPs. Crucially, none of the samples exceed a ΔE value of 2, the threshold above which color differences become noticeable to observers [57]. These findings indicate that cold plasma treatment had no recognizable effect on the color profile of the SCPs (Figure 1C).

### 3.4. Technofunctional Properties of SCPs

#### 3.4.1. Protein Solubility

To determine the intended application of proteins in food systems, its technofunctional properties, most importantly, solubility, must be characterized. The protein solubility of SCPs exhibited a typical U-shaped profile, reaching a minimum at the isoelectric point (around pH 4) and increasing under both more acidic and alkaline conditions (Figure 2A). The solubility under highly acidic conditions (pH 2) was even higher than under slightly alkaline conditions (pH 6 and 8), consistent with the findings by Fasuan et al. [42] and Nouska et al. [35]. This trend was not affected by the extraction method (*p* > 0.05), and this broad solubility profile expands the potential applications of SCPs across diverse food matrices, rendering them suitable for both acidic and alkaline formulations.

Conventionally extracted and ultrasound-pretreated SCPs exhibited a higher solubility than enzyme-pretreated SCP. At pH 10, C-C and U-C reached 100% solubility, whereas E-C achieved only 80.60%. Although an increased solubility was expected after enzymatic pretreatment, this observed reduction may stem from the co-precipitation of carbohydrates released during cell wall hydrolysis alongside the proteins, or from thermal and mild acidic denaturation occurring during the enzymatic incubation [14]. Given E-C’s considerably darker color compared to other SCPs (Figure 1C), a degree of denaturation likely occurred during enzymatic pretreatment (Figure 2A). Furthermore, the potential formation of potent protein-polyphenol complexes in E-C and E-P may have further restricted the protein solubility [58].

Cold plasma treatment can modulate the protein solubility in either direction depending on the physicochemical and structural properties, and process parameters [28]. Plasma can enhance solubility through multiple mechanisms, including the introduction of hydrophilic functional groups, partial unfolding that exposes buried hydrophilic residues and increases flexibility, surface etching and particle size reduction, and increased surface charge that enhances electrostatic repulsion and limits aggregation [28,30]. The improved solubility of C-P relative to non-treated C-C likely operates through these mechanisms. Conversely, the solubility of E-C, which was already lower than that of other SCPs, decreased further following cold plasma exposure. Excessive oxidative modifications can cause over-unfolding, which exposes too many hydrophobic amino acid residues that are normally hidden in the protein’s core [54]. The cold plasma treatment may have reduced the solubility due to this mechanism following the reactions that occurred during enzymatic pretreatment. Where a higher solubility is required, applying high-intensity ultrasound presents a viable option; a 6-min application has been reported to increase sesame cake protein solubility from 54.72% to 80.73% [9].

#### 3.4.2. Emulsifying Properties

The amphiphilic character and macromolecular structure of proteins impart interfacial activity, facilitating adsorption at the oil–water interface and the formation of interfacial films that reduce surface tension and prevent droplet aggregation [15]. The emulsifying activity and emulsion stability of plant proteins can be evaluated through turbidity, or droplet size and zeta potential. The size of the oil droplets in oil-in-water emulsions has a direct effect on the emulsion stability, where smaller sizes confer superior stability [16]. Emulsions with a zeta potential below −20 mV or above +20 mV generally maintain electrostatic stability, whereas values close to zero (−5 to +5 mV) are associated with rapid droplet aggregation [59]. Across both metrics, all emulsions stabilized by SCPs displayed a similar trend depending on the pH: the emulsifying activity increased and droplet size decreased as the pH shifted toward alkaline conditions (Figure 2B). Although the values for E-C and E-P were higher, the zeta potentials of all emulsions at pH 4 remained close to zero. At a pH value very close to the isoelectric point, both large droplet sizes and low zeta potentials indicate that the emulsion was unstable. This can be directly related to the solubility of the SCPs. A higher protein solubility increases the concentration of protein molecules available to adsorb at the oil–water interface, thereby promoting emulsion formation, whereas insufficient soluble protein weakens the electrostatic repulsion between dispersed oil droplets, and accelerates droplet aggregation [58].

The EAI of SCPs in this study was higher than [15], comparable to [11], or lower than [9,24] the values reported for sesame proteins in the literature. Hou et al. [37] reported extremely high EAI values, exceeding 200 m^2^/g across all conditions, which decreased as the duration of cold plasma treatment during extraction increased. Examining the effect of the extraction method on the emulsion properties revealed that SCPs obtained via enzymatic pretreatment differed markedly from others in terms of both the EAI and ESI (Figure 2). Although the zeta potential values remained similar across groups, SCPs obtained by applying enzymatic pretreatment formed emulsions with smaller droplet sizes. This behavior aligns with the discussions above: i) the color of the SCP produced by enzymatic pretreatment was much darker than others, suggesting the co-extraction of compounds like polysaccharides, which can delay the phase separation of the emulsion by increasing the viscosity of the continuous aqueous phase [15]; and ii) the brown color of the E-C was attributed to the partially oxidized polyphenols, which can interact with proteins to form protein–polyphenol complexes that enhance the emulsifying properties of the proteins [47].

Cold plasma treatment had a significant effect on the EAI, but not on the ESI. Similarly, the zeta potential did not differ among treated or untreated samples, but the droplet size was decreased, indicating an enhanced emulsion property (Figure 2C) (*p* < 0.05). Although this effect varied across extraction methods and pH levels, cold plasma exposure markedly improved the emulsifying properties in enzymatically pretreated SCP, especially in terms of the EAI. These differences likely stem from the varying effects of cold plasma on the protein structure, highlighting the need to optimize application times for each specific SCP. Moderate plasma treatment promotes partial protein unfolding and improves interfacial adsorption, whereas excessive oxidation induces protein aggregation, reducing the interfacial functionality and emulsifying properties [60]. It can enhance the interfacial activity by the loss of tertiary structure due to partial unfolding, thereby increasing the structural flexibility and facilitating protein adsorption and rearrangement at the oil–water interface, which contribute to a lower interfacial tension [52].

#### 3.4.3. Water and Oil Holding Capacities

The WHC and OHC of all SCPs were similar, indicating that the extraction method did not significantly affect these properties. The WHC values of SCPs were lower than [24], comparable to [35], or more than 2-fold higher than [8,9] the values reported for sesame proteins. A similar variation across the literature values was observed for the OHC. Among the SCPs, conventionally extracted and ultrasound-pretreated samples generally exhibited a higher WHC than OHC, a difference that was statistically significant except for C-P, suggesting a predominantly hydrophilic character. In contrast, enzyme-pretreated samples displayed a higher OHC than WHC, although this difference was not statistically significant. This shift may be attributed to a higher relative abundance of hydrophobic amino acids (including isoleucine, valine, glycine, and alanine) in the enzyme-treated samples (Table 1), which enhances the oil binding capacity. Furthermore, as the protein solubility typically correlates positively with the WHC [61], the lower WHC observed in enzyme-pretreated SCPs is consistent with their reduced solubility.

Cold plasma treatment did not significantly alter the WHC or OHC of the SCPs (Figure 2C). However, the slight increase in OHC observed following plasma treatment may stem from plasma-induced protein unfolding, which exposes hydrophobic amino acid residues and non-polar side chains, thereby promoting hydrophobic interactions with lipid molecules [30,54]. The balanced WHC and OHC retained across treatment enhances the versatility of SCPs for food formulations. Plant proteins exhibiting balanced hydration and lipophilic profile can be used as meat extenders or in plant-based meat analogs [62]. Additionally, they also play a crucial role in bakery and pasta products by improving the loaf volume and texture, and increasing the shelf life [61].

#### 3.4.4. Foaming Capacity and Stability

Similar to their emulsifying properties, the amphiphilic nature of proteins enables adsorption at interfaces, making them effective stabilizing agents for multiphase systems, including foams [52]. Previous studies have reported FC values for sesame protein around 60%, approximately one-third of our findings [8,35]. Although ultrasound treatment for 4 min increased the FC of SCP from 83.95% to 160.19% [9], these values were still lower than the SCPs in our study. On the other hand, Achouri et al. [11] reported nearly 2-fold higher FC values, using a completely different foaming protocol; nevertheless, the increment of FC as the pH increases aligns with our study.

Under acidic conditions, conventionally extracted SCPs exhibited the highest FC values, whereas the performance across extraction methods was comparable at other pH levels. While cold plasma treatment was not found to be effective on SCPs extracted with pretreatments, it significantly increased the FC of conventionally extracted SCPs (C-P compared to C-C). Plasma-induced oxidation causes partial protein denaturation, exposing hydrophobic residues and side chains that accelerate the formation of a stronger viscoelastic interfacial film and improve foaming properties [45]. Rout and Srivastav [29] attributed the 1.18–1.23 fold increase in FC following cold plasma treatment to the increased molecular flexibility as it unfolds. In contrast, the FC ultrasound- and enzyme-pretreated SCPs did not increase upon cold plasma treatment; in fact, it was observed to decrease at pH 6. Applying excessive cold plasma may induce over-oxidation and unfolding, promoting crosslinking and the formation of insoluble, large protein aggregates [54]. This mechanism aligns with our solubility data, where cold plasma enhanced solubility in conventionally extracted SCPs but impaired it in ultrasound- and enzyme-pretreated samples (Figure 2D). Nevertheless, plasma enhanced the FS across all extractions, under certain pH ranges and time intervals. Following adsorption at the air–water interface, cold plasma also induces mild oxidative cross-linking and enhances hydrophobic interactions, thereby preventing bubble coalescence and improving foam stability [28,29]. Proteins exhibiting a high foaming capacity can be used in bakery products, as they offer an alternative to animal proteins that increase volume and reduce baking loss [62].

### 3.5. Molecular Weight Distribution and Proteome Analysis

The dominant proteome differences across extraction methods and cold plasma treatment conditions were observed around ~40 kDa and ~20 kDa regions, which were revealed by SDS-PAGE analysis (Figure 3). Notably, reductions in the protein band intensity following cold plasma treatment were specifically pronounced in the ultrasound-pretreated samples (Figure 3), suggesting that ultrasound pretreatment may modulate the protein susceptibility of the plasma-induced modification by conferring a structural state. An LC-MS/MS analysis of the excised bands identified 11S globulin seed storage protein 2 (Q9XHP0; theoretical MW: 50.5 kDa) from band S1, and Peroxiredoxin-1 (Q06830; 25.4 kDa) and 60S ribosomal protein L12-3 (A0A6I9T1M7; 18.3 kDa) from band S2, all at a protein-level FDR of 5% with one unique peptide per identification (Table 6). The absence of all three identified proteins in the cold-plasma-treated sample aligns with plasma-induced oxidative structural disruption. Reactive oxygen and nitrogen species generated during cold plasma treatment oxidize the cysteine and methionine residues, disrupting the disulfide-linked subunit architecture of 11S globulins and promotes aggregation [54]. In agreement with this mechanism, the 11S globulin seed storage protein 2 detected by LC-MS/MS showed acidic subunits (~30–40 kDa) of sesame 11S globulin heterodimers in S1, but not basic subunits (~20–25 kDa) in S2 (Figure 3) [63]. The catalytic cysteine of Peroxiredoxin-1 is also intrinsically susceptible to irreversible hyperoxidation [64].

The absence of 11S globulin seed storage protein 2 in the cold-plasma-treated sample represents a significant clinical importance beyond structural modification. Since the 11S globulin family consists of clinically defined IgE-reactive sesame allergens (known as isoforms Ses i 6 and Ses i 7), 11S globulin seed storage protein 2 can trigger severe reactions, including anaphylaxis [65,66]. The IgE-binding epitopes of these isoforms provide the allergenic trait in relation to their 3D conformation. However, the conformation seems to be disrupted by plasma-generated reactive oxygen and nitrogen species. The oxidation of aromatic and sulfur-containing amino acid residues and the breakage of disulfide bonds lead proteins to unfold, ultimately affecting the final allergenicity [65,66]. Supporting this mechanism, a recent study demonstrated a 23% reduction in allergen-binding capacity by altering the IgE-binding epitopes of sesame proteins from sesame milk subjected to cold plasma budding [67]. These findings provide a molecular rationale for combining ultrasound pretreatment with cold plasma processing as an innovative strategy to develop hypoallergenic sesame cake protein ingredients.

## 4. Materials and Methods

### 4.1. Materials

The sesame cake was obtained from Tayf Bitkisel (Muğla, Türkiye) after the sesame seeds harvested from Gökova region were cold-pressed to produce sesame oil. Sesame cake was stored at 4 °C until use. Viscozyme (carbohydrase mixture) was gifted from Novonesis (Bagsvaerd, Denmark), and was used as received. All chemicals used were of analytical grade.

### 4.2. Extraction of Sesame Cake Proteins (SCPs)

Sesame cake was grounded and the remaining oil was removed as a pretreatment to increase extraction efficiency before protein extraction analyses. For defatting process, the grounded sesame cake was mixed with hexane at a ratio of 1:4 (*w*/*v*) and shaken at 250 rpm for 2 h at room temperature. At the end of this period, the mixture was centrifuged at 8000 rpm for 8 min, and the remaining cake was filtered through filter paper. After repeating the same process twice, the sesame cake was filtered through the filter paper and was dried in an oven at 30 °C for 1 d. After defatting, the fat content of sesame cake was decreased from 20.63 ± 1.12% to 2.29 ± 0.11%. In all protein extractions, this defatted sesame cake powder was used.

The protein content of defatted sesame cake and SCPs was determined by standard Kjeldahl method, using the protein conversion factor 6.25. The extraction conditions of conventional alkaline extraction, ultrasound pretreatment, and enzyme pretreatment were determined by preliminary studies, selecting the conditions with highest values in terms of yield, solubility, emulsion activity, and zeta potential.

#### 4.2.1. Conventional Alkaline Extraction

Defatted sesame cake powder was mixed with distilled water at a ratio of 1:10 (*w*/*v*). The pH of the mixture was adjusted to 9 using 1 M NaOH, and stirred at 1000 rpm for 3 h. Afterwards, the mixtures were centrifuged at 4000 rpm for 20 min. The solubilized proteins in the supernatant were precipitated by adjusting the pH to the isoelectric point (4.5) using 1 M HCl. After stirring for 10 min, it was centrifuged at 4000 rpm for 20 min. The precipitated proteins were collected and the pH was adjusted to 7. The protein fraction was freeze-dried and grounded into fine particles for further use.

#### 4.2.2. Ultrasound-Pretreated Alkaline Extraction

Defatted sesame cake powder was mixed with distilled water at a ratio of 1:10 (*w*/*v*). The mixture was placed in an ultrasonic water bath operating at 37 kHz and subjected to ultrasound at 96 W (40% power) for 15 min. At the end of ultrasonication, the conventional alkaline extraction was applied as described in Section 4.2.1.

#### 4.2.3. Enzyme-Pretreated Alkaline Extraction

Defatted sesame cake powder was mixed with distilled water at a ratio of 1:10 (*w*/*v*). The pH of the mixture was adjusted to 5 using 1 M HCl. For enzyme pretreatment, a carbohydrase mixture (Viscozyme, Novonesis) with a concentration of 300 µL/g sesame cake was added and the reaction mixtures were stirred at 250 rpm at 50 °C for 1 h. After inactivating the enzyme in a boiling water bath for 1 min, the conventional alkaline extraction was applied as described in Section 4.2.1.

### 4.3. Application of Dielectric Barrier Discharge (DBD) Cold Plasma

The dielectric barrier discharge cold plasma system consisted of two round stainless steel electrodes with a width of 120 mm and thickness of 4 mm. The electrode at the top was coupled with a glass dielectric barrier (thickness of 2 mm). Then, 1 g of SCP was spread onto the bottom of a glass Petri, and placed between two electrodes, which generates a gap of 16 mm. The cold plasma was applied at 30 kV for 30 min using a direct current (DC) power supply. Treated SCPs were coded and stored at −20 °C until analyses were performed.

### 4.4. Amino Acid Profile of SCPs

Then, 0.1 g of SCP was mixed with 10 mL of 6 M HCl and incubated at 100 °C for 16 h. After cooling in ice bath, hydrolyzed sample was neutralized by adding a 6 M NaOH solution. The volume was adjusted to 50 mL with distilled water. Next, 10 μL of diluted sample was mixed with 50 μL 0.4 N borate buffer, 10 μL ortho-phthaldialdehyde reagent, and 300 μL distilled water in vials. The detection was performed with high-performance liquid chromatography (Shimadzu, Kyoto, Japan) equipped with a fluorescence detector (JASCO FP-4025, Kyoto, Japan) and a C18 column (150 × 4.60 mm). The mobile phase, with a flow rate of 1 mL/min, was composed of solvent A, 40 mM NaH_2_PO_4_ buffer (pH = 7.8), and solvent B, acetonitrile:methanol:water (45:45:10). The detection was carried out in 338 nm. Calibration curves were generated with amino acid standard solution (AAS18, Sigma-Aldrich, Darmstadt, Germany).

### 4.5. Physicochemical Properties of SCPs

#### 4.5.1. Fourier-Transform Infrared (FTIR) Spectroscopy

The FTIR spectra of the SCP samples were recorded at room temperature in the mid-IR range (400–4000 cm^−1^) via a FTIR spectrometer (Shimadzu, Kyoto, Japan). Each spectrum was taken over 15 scans with a resolution of 4 cm^−1^. The results obtained were analyzed via Lab Solutions IR (version 2.21) and Origin Lab Pro 2024 software.

#### 4.5.2. Thermal Properties

Thermal characterization of SCPs was performed with a differential scanning calorimetry (DSC, Q10, TA Instruments, New Castle, DE, USA) with a nitrogen flow of 50 mL/min. An empty hermetic pan was used as a reference. SCP sample was weighed in a hermetic pan and sealed with the appropriate lids via a sample encapsulation press (TA Instruments, USA). The temperature scans were performed from 0 °C to 200 °C at 10 °C/min. The data were collected and analyzed via software (TA Universal Analysis, version 4.5).

#### 4.5.3. Color

The color parameters (L*, a*, and b*) of SCP were determined using a hand colorimeter (Minolta Chroma Meter CR-400, Minolta Co., Ltd., Tokyo, Japan). The color change (ΔE) caused by cold plasma treatment was calculated with Equation (1).

(1) $$\Delta E={\left({\left(L^{*}-{L_{0}}^{*}\right)}^{2}+{\left(a^{*}-{a_{0}}^{*}\right)}^{2}+{\left(b^{*}-{b_{0}}^{*}\right)}^{2}\right)}^{0.5}$$

### 4.6. Technofunctional Properties of SCPs

#### 4.6.1. Protein Solubility

The protein solubility of SCPs was determined in a wide pH range between 2–10. SCP was mixed with distilled water at a concentration of 1% (*w*/*v*), and the pH was adjusted to desired point by using 1 M NaOH or 1 M HCl. Mixture was shaken at 250 rpm for 30 min, and hydrated overnight at 4 °C. Afterwards, the mixture consisting of dissolved proteins was centrifuged at 4000 rpm for 8 min to separate it from the insoluble protein precipitate. The amount of soluble protein in the mixture was determined spectrophotometrically using the Bradford method. In each well, 50 µL of the sample was mixed with 150 µL of Bradford reagent (B6916, Sigma–Aldrich, Darmstadt, Germany). For control, 50 µL distilled water was mixed instead of the protein solution. After storing in dark for 10 min, the absorbance of samples was measured at 595 nm by a microplate reader (Epoch 2, BioTek Instruments, Winooski, VT, USA). The calibration curve was generated using bovine serum albumin (BSA) and the results were expressed as mg BSA/g SCP. The solubility of SCPs were calculated with Equation (2).

(2) $$Protein\;solubility(\%)=\frac{Soluble\;protein\;content\;in\;different\;pH\;solutions}{Soluble\;protein\;content\;in\;1\;M\;NaOH\;solution}$$

#### 4.6.2. Emulsifying Properties

Then, 1% (*w*/*v*) protein solution at pH 4, 6, and 8 was prepared as described in Section 4.6.1. To prepare oil-in-water emulsions, corn oil was added to the protein solutions at a ratio of 4:1 (*v*/*v*) and homogenized using UltraTurrax (T25 Digital, IKA, Staufen, Germany) at 15,000 rpm for 2 min. To determine the emulsion activity index (EAI), 30 µL of the homogenized emulsion was mixed with 3 mL of 0.1% sodium dodecyl sulfate (SDS) solution. The absorbance of the mixed solution at 500 nm was measured using a microplate reader. For the emulsion stability index (ESI), a sample was taken 10 min after emulsion formation. The EAI and ESI were calculated according to Equations (3) and (4), respectively:

(3) $$EAI\;(m^{2}/g)=\frac{2\times 2.303\times A_{0}\times d}{C\times \Phi \times 10^{4}}$$

(4) $$ESI\;(min)=\frac{A_{0}\times 10}{A_{0}-A_{10}}$$

where A_0_ represents the absorbance at 500 nm right after homogenization; A_10_ represents the absorbance at 500 nm 10 min after homogenization; d represents the dilution factor; C represents the initial protein concentration (g/mL); and Φ represents the oil content in the emulsion.

The droplet size (diameter, nm) and zeta potential (mV) of oil droplets in oil-in-water emulsion samples were measured by dynamic light scattering (DLS) equipment (Malvern Zetasizer NanoZS, Worcestershire, UK). Refractive index of the corn oil and water were used as 1.473 and 1.330, respectively.

#### 4.6.3. Water and Oil Holding Capacities

Both water and oil holding capacity (WHC and OHC) of SCP were determined gravimetrically. Then, 0.1 g SCP was mixed with 3 mL of distilled water and corn oil for WHC and OHC, respectively. The mixture was vortexed for 1 min and left in room temperature for 30 min. Afterwards, the mixture was centrifugated at 4000 rpm for 20 min. The pellet and the supernatant were weighed, and the amount of water or oil absorbed by the SCP was calculated. The results were expressed as g water or oil/g SCP.

#### 4.6.4. Foaming Capacity and Stability

SCP was mixed with distilled water at a concentration of 20 mg/mL, and the pH of the solution was adjusted to 4, 6, and 8 by using 1 M NaOH or 1 M HCl. The mixture was homogenized at 20,000 rpm for 1 min by using UltraTurrax (T25 Digital, IKA, Staufen, Germany). The foaming capacity (FC) and foam stability (FS) were calculated according to Equations (5) and (6), respectively:

(5) $$FC\;(\%)=\frac{V_{1}-V_{0}}{V_{0}}\times 100$$

(6) $$FS\;(\%)=\frac{V_{2}-V_{0}}{V_{1}-V_{0}}\times 100$$

where V_0_ represents the volume of the solution before homogenization; V_1_ represents the volume right after the homogenization; and V_1_ represents the volume 10, 30, and 60 min after the homogenization.

### 4.7. Molecular Weight Distribution (SDS-PAGE)

Next, 0.2 g of SCP was redissolved in distilled water with occasional vortexing and sonicated for 30 min at 37 °C (20% amplitude) by using sonicator (Hydraultrasonic, Istanbul, Turkey). Then, the extracts from samples were cleared at 15,000 rpm for 15 min at room temperature. The resulting supernatant, containing the soluble protein fraction, was carefully transferred to a new microcentrifuge tube and stored at −20 °C [68].

To resolve proteins as bands, Sodium Dodecyl Sulfate-Polyacrylamide Gel Electrophoresis (SDS-PAGE) was applied using the Tris-Tricine-SDS running buffer at a constant voltage of 100 V at RT. Afterwards, SDS-polyacrylamide gels were stained with Coomassie Brilliant Blue R-250 solution (Sigma–Aldrich, Darmstadt, Germany). Gel images were acquired using an iBright CL750 Imaging System (Thermo Fisher Scientific, Waltham, MA, USA).

### 4.8. Mass-Spectrometry-Based Proteomics

Protein bands of interest were cut from the SDS-polyacrylamide gel, and then reduction/alkylation of these was performed following the protocol in the Pierce™ Mass Spec Sample Prep Kit (Thermo Fisher Scientific, Waltham, MA, USA). Briefly, the gel pieces were destained (25 mM NH_4_HCO_3_ in 50% acetonitrile) and reduced (10 mM dithiothreitol) for 45 min at 50 °C. This step was followed by the alkylation (50 mM iodoacetamide) of the protein samples for 20 min at RT in the dark. Corresponding peptides for each of these protein samples were prepared by adding MS-grade trypsin and incubating for 16 h at 37 °C. The peptide samples were cleaned by applying ZipTip™ C18 (Thermo Fisher Scientific, Waltham, MA, USA) before injecting into LC-MS/MS. Then, peptides were separated on the Thermo Ultimate 3000 RSLCnano system (Thermo Fisher Scientific, Waltham, MA, USA) using a 120 min gradient elution, followed by analysis with the Thermo Q Exactive mass spectrometer (Thermo Fisher Scientific, Waltham, MA, USA). The raw data were processed in comparison to each other (cold-plasma-treated and non-treated) through data-dependent acquisition (DDA) nano-LC-MS/MS. Following mass spectrometry, database searches were conducted against the *Sesamum indicum* (taxon 4182) reference proteome using FragPipe (MSFragger v4.4.1; IonQuant (version 1.5.5, Ann Arbor, MI, USA); Percolator (version 3.09, University of Washington, Seattle, WA, USA and Stockholm University, Sweden), with label-free quantification with match-between-runs and protein-level false discovery rate at 5%. Meanwhile, differential analysis at the band level was performed in R using the DEqMS algorithm. To validate the selected peptide-spectrum matches, annotated MS/MS spectra with *b*- and *y*-ion assignments were generated in Python (version 3.10) using the pyteomics and spectrum_utils libraries.

### 4.9. Statistical Analysis

All experiments were analyzed statistically via Minitab (version 18.1, LLC, State College, PA, USA). Each experiment was conducted as three replicates, and the results were given as mean ± standard deviation. The differences between groups were compared via general linear model followed by Tukey’s multiple-range test. Differences were considered significant if *p* < 0.05.

## 5. Conclusions

The present study evaluated the upcycling of sesame seed cake into plant-based proteins, an approach already recognized as efficient, by investigating strategies to modify their technofunctional properties. The extraction method, whether conventional alkaline extraction or its combination with ultrasound or enzymatic pretreatments, significantly influenced multiple technofunctional attributes of the resulting SCPs. Post-extraction cold plasma treatment induced structural modifications that further altered these properties. Importantly, the responsiveness of SCPs to cold plasma exposure was shown to depend on the extraction method employed, revealing an extraction-dependent plasma sensitivity that has not been previously documented. Proteomic analysis revealed that cold plasma treatment particularly downregulated 11S globulin seed storage protein 2, a primary allergenic component of sesame, thereby providing mechanistic evidence for the reduced allergenicity. Collectively, these results highlight cold plasma treatment as a promising tool to enhance technofunctional properties while mitigating the allergenic potential of sesame proteins, broadening their applicability as sustainable food ingredients.

To the best of our knowledge, this is the first comparative study evaluating the interactive effects of cold plasma treatment alongside pretreatments applied prior to protein extraction. Future studies should focus on extending this combined approach to other plant protein sources to determine whether extraction-dependent plasma sensitivity represents a universal phenomenon. Furthermore, exploring the underlying molecular mechanisms via advanced structural tools, evaluating the functionality and digestibility of plasma-treated proteins in real food systems, and conducting pilot-scale trials with technoeconomic assessments will facilitate the development of sustainable plant protein ingredients tailored for food and biotechnological applications.

## Figures and Tables

**Table 1:** Amino acid concentrations (mg/g) of sesame cake proteins (SCPs).

Amino Acid	C-C	C-P	U-C	U-P	E-C	E-P
Aspartic Acid	61.80 ± 2.05	54.84 ± 1.24	64.06 ± 5.97	57.42 ± 1.16	61.57 ± 2.36	66.14 ± 1.34
Glutamic Acid	125.94 ± 3.68	109.26 ± 0.71	130.37 ± 14.35	114.96 ± 1.57	141.48 ± 27.94	137.45 ± 4.44
Serine	36.43 ± 8.74	29.37 ± 5.43	45.24 ± 0.05	39.65 ± 0.96	38.86 ± 2.65	36.36 ± 6.22
Histidine	12.71 ± 2.12	11.05 ± 1.23	13.37 ± 1.53	12.08 ± 1.41	12.43 ± 1.98	11.82 ± 0.67
Glycine	14.74 ± 9.51	11.56 ± 7.16	13.63 ± 0.82	11.77 ± 1.18	15.97 ± 3.61	10.26 ± 2.19
Threonine	49.30 ± 16.78	37.30 ± 10.24	59.01 ± 0.30	38.86 ± 8.25	58.69 ± 6.50	56.08 ± 5.81
Arginine	92.16 ± 7.89	79.13 ± 4.50	93.65 ± 6.96	88.64 ± 4.39	102.34 ± 12.32	96.75 ± 4.68
Alanine	48.27 ± 1.88	42.03 ± 1.55	48.14 ± 2.21	44.10 ± 1.26	57.16 ± 9.80	50.47 ± 3.25
Tyrosine	42.78 ± 3.51	37.74 ± 3.06	41.98 ± 0.91	41.02 ± 4.24	38.86 ± 2.47	43.19 ± 1.21
Cystine	56.25 ± 23.61	56.54 ± 8.95	32.10 ± 1.47	64.32 ± 1.18	77.56 ± 14.46	66.43 ± 2.04
Valine	43.79 ± 2.03	39.11 ± 1.10	45.48 ± 2.98	45.25 ± 3.10	50.09 ± 7.71	39.97 ± 0.35
Methionine	75.32 ± 5.66	68.34 ± 3.11	69.51 ± 2.07	66.21 ± 1.11	69.59 ± 1.94	77.48 ± 5.26
Phenylalanine	22.42 ± 0.98	20.91 ± 0.70	22.26 ± 1.92	22.99 ± 0.64	25.48 ± 2.64	22.41 ± 2.74
Isoleucine	80.66 ± 7.12	68.92 ± 3.06	74.80 ± 0.48	73.92 ± 5.29	76.90 ± 5.30	81.89 ± 5.72
Leucine	103.28 ± 4.89	89.26 ± 1.18	118.56 ± 0.19	91.74 ± 3.79	91.63 ± 1.79	101.74 ± 6.04
Lysine	39.36 ± 7.61	37.66 ± 0.34	42.93 ± 11.56	44.04 ± 6.99	35.74 ± 5.32	34.17 ± 3.83
Total EAA	426.82 ± 20.66	372.55 ± 14.74	439.85 ± 2.19	398.94 ± 14.12	419.42 ± 18.47	425.10 ± 27.60
Total AA	905.18 ± 34.30	793.02 ± 29.44	914.67 ± 41.09	856.97 ± 50.47	954.37 ± 19.52	932.60 ± 43.22

EAA: Essential amino acids (histidine, threonine, valine, methionine, phenylalanine, isoleucine, leucine, and lysine). AA: Amino acids.

**Table 2:** Thermal degradation characteristics of SCPs.

SCP	T_start_ (°C)	T_onset_ (°C)	T_max_ (°C)	T_stop_ (°C)
C-C	74.32 ± 6.20 ^D^	79.18 ± 7.30 ^D^	123.47 ± 6.04 ^D^	178.33 ± 4.05 ^B^
C-P	151.33 ± 0.87 ^A^	154.62 ± 0.40 ^A^	170.01 ± 0.42 ^A^	194.36 ± 3.85 ^AB^
U-C	118.12 ± 3.38 ^BC^	121.24 ± 5.59 ^BC^	147.50 ± 2.18 ^BC^	187.24 ± 3.97 ^AB^
U-P	98.65 ± 4.19 ^C^	102.06 ± 0.17 ^CD^	139.04 ± 5.64 ^CD^	185.69 ± 2.60 ^AB^
E-C	130.72 ± 7.69 ^AB^	135.95 ± 10.47 ^AB^	160.59 ± 6.12 ^AB^	205.20 ± 11.52 ^A^
E-P	104.07 ± 7.52 ^C^	111.25 ± 11.85 ^BC^	136.91 ± 1.03 ^CD^	188.97 ± 3.18 ^AB^

Different uppercase letters within the same column indicate significant differences (*p* < 0.05).

**Table 3:** The droplet size (Z-average and number mean, d.nm) and zeta potential of oil-in-water emulsions prepared with sesame cake proteins (SCPs) in different pH values.

SCP	pH	Z-Average (d.nm)	Number Mean (d.nm)	Zeta Potential (mV)
C-C	4	5451.00 ± 435.58 ^Ba^	523.45 ± 24.40 ^Ba^	2.38 ± 0.05 ^ABa^
	6	1114.78 ± 56.92 ^Bb^	363.30 ± 30.41 ^Bb^	−20.72 ± 1.39 ^ABb^
	8	531.20 ± 23.62 ^Bc^	148.73 ± 1.17 ^Bc^	−26.20 ± 1.08 ^ABc^
C-P	4	4104.67 ± 375.24 ^Ba^	321.33 ± 23.44 ^Ba^	2.22 ± 0.07 ^ABa^
	6	1015.93 ± 137.99 ^Bb^	259.83 ± 2.88 ^Bb^	−20.18 ± 1.87 ^ABb^
	8	586.98 ± 3.18 ^Bc^	179.75 ± 14.88 ^Bc^	−20.70 ± 1.65 ^ABc^
U-C	4	5380.00 ± 100.41 ^Aa^	433.40 ± 34.80 ^Aa^	1.96 ± 0.05 ^Ba^
	6	1067.98 ± 69.33 ^Ab^	399.08 ± 35.76 ^Ab^	−20.38 ± 2.30 ^Bb^
	8	731.60 ± 28.92 ^Ac^	226.10 ± 21.92 ^Ac^	−23.19 ± 1.50 ^Bc^
U-P	4	4922.42 ± 107.60 ^Aa^	426.50 ± 34.65 ^Aa^	0.92 ± 0.15 ^Ba^
	6	1197.00 ± 43.84 ^Ab^	253.69 ± 11.09 ^Ab^	−22.55 ± 1.53 ^Bb^
	8	785.35 ± 2.47 ^Ac^	150.07 ± 19.04 ^Ac^	−23.40 ± 2.26 ^Bc^
E-C	4	1687.00 ± 126.68 ^Ca^	460.85 ± 13.51 ^Ba^	6.59 ± 0.13 ^Aa^
	6	1162.93 ± 128.80 ^Cb^	357.98 ± 30.58 ^Bb^	−20.05 ± 2.19 ^Ab^
	8	761.30 ± 29.84 ^Cc^	207.21 ± 14.37 ^Bc^	−24.88 ± 2.23 ^Ac^
E-P	4	1521.33 ± 81.55 ^Ca^	315.72 ± 13.74 ^Ba^	8.57 ± 0.35 ^Aa^
	6	1114.97 ± 110.83 ^Cb^	294.45 ± 23.97 ^Bb^	−22.25 ± 1.72 ^Ab^
	8	699.95 ± 39.95 ^Cc^	121.71 ± 7.34 ^Bc^	−25.22 ± 1.48 ^Ac^

Different uppercase letters within the same column indicate significant differences among extraction methods at the same pH value (*p* < 0.05). Different lowercase letters within the same column indicate significant differences across pH values for the same extraction method (*p* < 0.05).

**Table 4:** Water and oil holding capacities of sesame cake proteins (SCPs).

SCP	WHC (g Water/g SCP)	OHC (g Oil/g SCP)
C-C	2.91 ± 0.13 *	2.38 ± 0.12
C-P	2.57 ± 0.25	2.88 ± 0.17
U-C	2.81 ± 0.02 *	1.93 ± 0.15
U-P	2.83 ± 0.05 *	2.05 ± 0.15
E-C	2.39 ± 0.21	2.58 ± 0.09
E-P	2.45 ± 0.13	2.70 ± 0.18

* indicates a significant difference between the WHC and OHC of the same SCP (*p* < 0.05).

**Table 5:** The foam stability (FS) of SCPs in the pH range of 4–8.

SCP	pH	FS_10_ (%)	FS_30_ (%)	FS_60_ (%)
C-C	4	61.11 ± 1.57 ^Ca^	40.00 ± 0.00 ^Ba^	25.22 ± 4.09 ^Ca^
	6	48.89 ± 4.64 ^Cb^	41.62 ± 4.64 ^Bb^	29.39 ± 1.15 ^Cb^
	8	84.74 ± 1.99 ^Ca^	71.92 ± 2.72 ^Ba^	65.64 ± 1.45 ^Cc^
C-P	4	97.92 ± 2.95 ^Ca^	85.67 ± 3.30 ^Ba^	79.58 ± 0.59 ^Ca^
	6	96.04 ± 0.78 ^Cb^	84.93 ± 0.78 ^Bb^	71.45 ± 1.60 ^Cb^
	8	89.38 ± 3.42 ^Ca^	84.81 ± 3.03 ^Ba^	63.45 ± 4.55 ^Cc^
U-C	4	100.00 ± 0.00 ^Aa^	72.50 ± 3.54 ^Aa^	50.00 ± 0.00 ^Aa^
	6	95.00 ± 1.93 ^Ab^	81.69 ± 1.93 ^Ab^	71.69 ± 1.69 ^Ab^
	8	98.37 ± 0.17 ^Aa^	88.80 ± 3.44 ^Aa^	77.23 ± 0.06 ^Ac^
U-P	4	100.00 ± 0.00 ^Aa^	90.00 ± 14.14 ^Aa^	80.00 ± 0.00 ^Aa^
	6	89.60 ± 5.65 ^Ab^	79.34 ± 5.65 ^Ab^	72.60 ± 3.39 ^Ab^
	8	91.44 ± 2.67 ^Aa^	83.12 ± 0.65 ^Aa^	73.15 ± 2.14 ^Ac^
E-C	4	97.50 ± 3.54 ^Ba^	82.50 ± 3.54 ^Ba^	67.50 ± 3.54 ^Ba^
	6	78.02 ± 1.43 ^Bb^	74.85 ± 1.43 ^Bb^	69.19 ± 2.12 ^Bb^
	8	92.45 ± 5.96 ^Ba^	65.69 ± 1.39 ^Ba^	48.53 ± 2.08 ^Bc^
E-P	4	100.00 ± 0.00 ^Ba^	100.00 ± 0.00 ^Ba^	97.50 ± 3.54 ^Ba^
	6	60.21 ± 2.65 ^Bb^	43.14 ± 2.65 ^Bb^	39.12 ± 1.25 ^Bb^
	8	94.35 ± 2.10 ^Ba^	65.10 ± 2.22 ^Ba^	49.27 ± 3.00 ^Bc^

Different uppercase letters within the same column indicate significant differences among extraction methods at the same pH value (*p* < 0.05). Different lowercase letters within the same column indicate significant differences across pH values for the same extraction method (*p* < 0.05).

**Table 6:** Identification results for the proteins by LC-MS/MS analyses.

Sample	Prot. ID	Prot. Description	MW (kDa)	Pep. No	Pep. Seq.
S1	Q9XHP0	11S globulin seed storage protein 2	50.5	1	GSQSFLLSPGGR
S2	Q06830	Peroxiredoxin-1	25.4	1	ADEGISFR
S2	A0A6I9T1M7	60S ribosomal protein L12-3	18.3	1	IGPLGLSPK

## Data Availability

The dataset is available upon request from the authors.

## Abbreviations

SCP	Sesame cake protein
C-C	Non-plasma-treated (control) conventionally alkaline-extracted SCP
C-P	Plasma-treated conventionally alkaline-extracted SCP
U-C	Non-plasma-treated (control) ultrasound-pretreated alkaline-extracted SCP
U-P	Plasma-treated ultrasound-pretreated alkaline-extracted SCP
E-C	Non-plasma-treated (control) enzyme-pretreated alkaline-extracted SCP
E-P	Plasma-treated enzyme-pretreated alkaline-extracted SCP
